# Predictors of lipoprotein(a) variability in clinical practice and their impact on cardiovascular risk

**DOI:** 10.1186/s12944-025-02666-8

**Published:** 2025-07-23

**Authors:** Hyung Joon Joo, Seung Gyu Yun, Jae Hyoung Park, Soon Jun Hong, Cheol Woong Yu, Seung Yong Shin, Eung Ju Kim

**Affiliations:** 1 Department of Cardiology, Korea University Anam Hospital, 73, Inchon-ro, Seongbuk-gu, Seoul, 02841 Republic of Korea; 2 Department of Medical Informatics, Korea University College of Medicine, Seoul, Republic of Korea; 3 Department of Laboratory Medicine, Korea University Anam Hospital, Seoul, Republic of Korea; 4 Department of Cardiology, Korea University Ansan Hospital, Ansan, Republic of Korea; 5 Department of Cardiology, Korea University Guro Hospital, Seoul, Republic of Korea

**Keywords:** Lipoprotein(a), Variability, Cardiovascular risk, Risk stratification

## Abstract

**Background:**

Lipoprotein(a) (Lp[a]) is an established cardiovascular risk marker; however, its intraindividual variability and implications for risk stratification remain poorly understood. This study investigated the clinical and biochemical predictors of high Lp(a) levels and evaluated their potential roles in cardiovascular risk assessment to inform evidence-based public health strategies for cardiovascular disease prevention.

**Methods:**

This retrospective multicenter observational study was conducted using data from three tertiary university hospitals in Korea. Patients with at least two Lp(a) measurements taken ≥ 90 days apart were included (*n* = 5,305). High Lp(a)-level variability was defined as an absolute change of > 10 mg/dL and a relative change of > 25%. Predictors of high-variability were identified through regression analyses, and risk reclassification across Lp(a) risk categories was performed.

**Results:**

Baseline and follow-up Lp(a) levels were strongly correlated (*r* = 0.89, *P* < 0.01); however, substantial individual variability was observed, with a median absolute change of 3.9 mg/dL and a median percentage change of 26.3%. Approximately 19.9% of the patients exhibited high Lp(a) level variability, which was associated with lower baseline Lp(a) levels and higher follow-up Lp(a) levels, lower body mass indices, higher hemoglobin levels, elevated white blood cell and platelet counts, increased serum glucose levels, lower high-density lipoprotein cholesterol levels, and use of antihypertensive medications. Notably, risk reclassification analysis revealed marked variability among patients in the intermediate “gray-zone.”

**Conclusions:**

The findings of this study indicate that Lp(a) level variability is associated with adverse cardiovascular risk profiles and dynamic risk reclassification. These results highlight the potential of serial Lp(a) measurements to refine cardiovascular risk stratification, particularly in intermediate-risk patients. Integrating these findings into clinical practice guidelines has the potential to improve cardiovascular risk management at the population level, reduce healthcare disparities, and inform targeted public health interventions aimed at cardiovascular prevention.

**Supplementary Information:**

The online version contains supplementary material available at 10.1186/s12944-025-02666-8.

## Background

Lipoprotein(a) (Lp[a]) is a plasma lipoprotein that is increasingly recognized as an independent cardiovascular risk factor. Elevated Lp(a) levels are associated with an increased risk of atherosclerotic cardiovascular diseases, including myocardial infarctions, strokes, and peripheral arterial disease [1, 2]. Unlike other lipid fractions, Lp(a) levels are primarily genetically determined and are less influenced by lifestyle factors such as diet or exercise [3]. However, recent evidence suggests that Lp(a) levels may not be static and that variability in Lp(a) levels over time could have important clinical implications for cardiovascular risk assessment and management [4]. 

The clinical significance of Lp(a) level variability remains underexplored, with only a few studies addressing its potential impact on cardiovascular outcomes. Evidence indicates that fluctuations in Lp(a) levels could be associated with changes in inflammatory status, kidney function, or the use of lipid-lowering therapies, particularly proprotein convertase subtilisin/kexin type 9 (PCSK9) inhibitors, which have been shown to effectively reduce Lp(a) levels [5–7]. Another study reported that intraindividual variability in Lp(a) levels was up to 24.9%, and individuals with higher Lp(a) level variability may have a distinct cardiovascular risk profile compared to those with stable Lp(a) levels [8]. These findings highlight the need for a deeper understanding of the factors contributing to Lp(a) level variability and their implications for patient management.

Moreover, Lp(a) levels differ across racial and ethnic groups, with individuals of African descent and South Asian populations generally having higher median Lp(a) levels than Caucasian or East Asian individuals [9]. These differences are largely attributable to genetic factors, with variations in the KIV copy number and single-nucleotide polymorphisms in the LPA gene contributing to the observed disparities [5]. Among Asian subgroups, Asian Indian individuals demonstrate a higher prevalence of elevated Lp(a) levels (≥ 50 mg/dL) at 35.7%, compared to Chinese individuals who have the lowest prevalence at 31.2% [10]. This raises questions regarding the potential differences in Lp(a) level variability patterns and their clinical implications across diverse racial and ethnic populations. Furthermore, the association between Lp(a) levels and cardiovascular outcomes may differ among these subgroups, emphasizing the need for race-specific investigations of Lp(a) variability.

This study aimed to identify the clinical and biochemical predictors of high Lp(a) level variability in a large cohort of patients from multiple centers in Korea. The association between Lp(a) level variability and traditional cardiovascular risk factors was examined to clarify the potential implications of Lp(a) level variability in cardiovascular risk stratification and to characterize the clinical features of patients with high Lp(a) level variability.

## Methods

### Study design and data sources

This multicenter retrospective observational study used data from the Observational Medical Outcomes Partnership Common Data Model (OMOP-CDM) and hospital information systems of three tertiary university hospitals in South Korea. The OMOP-CDM framework, provided by the Observational Health Data Sciences and Informatics collaboration, serves as a standardized structure for organizing electronic health records. Clinical data, including diagnoses based on the International Classification of Diseases (10th revision) codes, medications, and laboratory results, were mapped to unique concept identifiers in the OMOP-CDM. All data were stored on Microsoft SQL servers and accessed using direct SQL queries.

### Study population

The study included patients who had Lp(a) level measurements taken twice with an interval of at least 90 days between January 1, 2019, and June 30, 2024. A total of 7,175 patients were initially identified, of whom 1,870 were excluded because of anomalous or missing data regarding baseline lipid profiles, liver function tests, or serum creatinine levels. The final study population included 5,305 patients.

### Serum lipoprotein(a) level measurement

The Lp(a) level was measured as part of routine clinical care using the Randox Lp(a) assay (Catalog No. LP3403; Randox Laboratories, Crumlin, United Kingdom) on a Beckman Coulter AU5800 analyzer. This immunoturbidimetric assay utilizes a polyclonal antibody against apolipoprotein(a) to minimize isoform bias, with a five-point calibration curve traceable to World Health Organization/International Federation of Clinical Chemistry and Laboratory Medicine Secondary Reference Material 2B. Despite the eight calibrator lot changes during the study, the daily internal controls maintained assay precision, with intra- and inter-assay coefficients of variation between 2.4% and 3.2%. The results were reported as milligrams per deciliter (mg/dL). Lp(a) level risk categories were defined based on the EAS consensus statement: high-risk (≥ 50 mg/dL or ∼125 nmol/L), low-risk (< 30 mg/dL or ∼75 nmol/L), and intermediate-risk (‘gray-zone’) for values between 30 and 50 mg/dL [5]. Lp(a) variability was assessed by calculating both the absolute and relative changes between the first and second measurements. High Lp(a) level variability was defined as an absolute change of > 10 mg/dL and a relative change of > 25%, based on thresholds adopted from previous studies, including the UK Biobank, IONIS-APO(a)Rx, and IONIS-APO(a)-LRx trials [11, 12]. 

### Definitions of covariates

Hypertension was defined as systolic blood pressure ≥ 140 mmHg, diastolic blood pressure ≥ 90 mmHg, or the use of antihypertensive drugs. Diabetes mellitus was defined as HbA1c levels ≥ 6.5%, fasting glucose levels ≥ 126 mg/dL, or the use of antidiabetic medications. Dyslipidemia was defined as a total cholesterol level ≥ 240 mg/dL, low-density lipoprotein (LDL) cholesterol level ≥ 160 mg/dL, triglyceride level ≥ 200 mg/dL, high-density lipoprotein (HDL) cholesterol level < 40 mg/dL, or use of lipid-lowering medications. Chronic kidney disease was defined as an estimated glomerular filtration rate (eGFR) < 60 mL/min/1.73 m² using the Chronic Kidney Disease Epidemiology Collaboration equation. Other medical histories, such as liver disease, thyroid disease, prior myocardial infarction, stroke, and heart failure, were identified using the OMOP-CDM concept IDs.

### Statistical analyses

Categorical variables were reported as frequencies and percentages, whereas continuous variables were presented as means with standard deviations. The normality of continuous variables was assessed using the Shapiro–Wilk test. Based on the normality of the results, comparisons between groups were performed using Student’s t-test or Mann–Whitney U test, as appropriate. Categorical variables were analyzed using chi-squared or Fisher’s exact tests. Spearman’s rank correlation coefficient was used to assess the relationships between continuous variables. Wilcoxon signed-rank test was used to compare the magnitudes of variability. To control for the risk of type I errors because of multiple comparisons (Table 1), Bonferroni correction was applied to the 40 variables examined, yielding a corrected significance threshold of 0.05/40 (approximately 0.00125). Only *P*-values below this threshold were considered statistically significant.

**Table 1:** Clinical characteristics of patients between high and low Lp(a) variability

	Low Lp(a) Variability (*N* = 4,248)	High Lp(a) Variability (*N* = 1,057)	Adjusted *P* Value
Demographic findings			
Age, years	62.5 ± 14.7	65.2 ± 13.1	< 0.001
Male	2438(57.4%)	618(58.6%)	1.00
Smoking	2214(52.1%)	570(53.9%)	1.00
Drinking	2522(59.4%)	636(60.2%)	1.00
Low SES	378(8.9%)	118(11.2%)	1.00
Body mass index	24.7 ± 3.7	24.4 ± 3.8	1.00
Hypertension	3182(74.9%)	891(84.3%)	< 0.001
Diabetes mellitus	1936(45.6%)	573(54.2%)	< 0.001
Dyslipidemia	3564(83.9%)	959(90.7%)	< 0.001
Chronic kidney disease	393(9.3%)	140(13.2%)	0.018
Liver Disease	582(13.7%)	158(14.9%)	1.00
Thyroid Disease	502(11.8%)	137(13.0%)	1.00
Prior myocardial infarction	257(6.0%)	79(7.5%)	1.00
Prior stroke	1066(25.1%)	343(32.5%)	< 0.001
Prior heart failure	226(5.3%)	76(7.2%)	1.00
Laboratory findings			
Hemoglobin, g/dL	13.5 ± 1.7	13.2 ± 1.9	< 0.001
WBC count (×1000/𝜇L)	6.7 ± 2.3	7.2 ± 2.7	< 0.001
Platelet count (×1000/𝜇L)	229.1 ± 70.7	233.3 ± 73.0	1.00
BUN, mg/dL	16.2 ± 6.7	17.3 ± 8.6	0.007
Creatinine, mg/dL	0.9 ± 0.8	1.0 ± 0.9	1.00
- eGFR, mL/min/1.73 m^2^	90.6 ± 21.7	87.4 ± 23.8	0.002
Total cholesterol, mg/dL	167.6 ± 45.1	169.2 ± 48.3	1.00
LDL cholesterol, mg/dL	98.2 ± 37.6	99.8 ± 41.5	1.00
HDL cholesterol, mg/dL	49.9 ± 13.4	48.1 ± 14.1	0.007
Triglyceride, mg/dL	126.4 ± 93.9	136.7 ± 101.0	0.111
Glucose, mg/dL	114.9 ± 37.4	123.5 ± 47.6	< 0.001
AST, unit/L	30.1 ± 39.8	32.9 ± 52.3	1.00
ALT, unit/L	25.4 ± 23.6	25.8 ± 35.5	1.00
Total bilirubin, mg/dL	0.7 ± 0.4	0.7 ± 0.4	1.00
Albumin, g/dL	4.14 ± 0.41	4.05 ± 0.48	< 0.001
Baseline Lp(a), mg/dL	21.4 ± 26.4	41.0 ± 34.3	< 0.001
- measured during hospitalization	967(22.8%)	218(20.6%)	1.00
Follow-up Lp(a), mg/dL	21.7 ± 26.2	53.2 ± 40.7	< 0.001
- measured during hospitalization	839(19.8%)	174(16.5%)	0.446
Medications			
Antiplatelet drugs	2332(54.9%)	678(64.1%)	< 0.001
Antihypertensive drugs	2670(62.9%)	787(74.5%)	< 0.001
Antidiabetic drugs	1347(31.7%)	411(38.9%)	< 0.001
Statin	2908(68.5%)	822(77.8%)	< 0.001
Ezetimibe	1186(27.9%)	365(34.5%)	0.002
PCSK9 inhibitor	28(0.7%)	16(1.5%)	1.00

Continuous variables are presented as means ± standard deviations, and categorical variables are presented as counts (percentages). To account for multiple comparisons, a Bonferroni correction was applied, yielding a significance threshold of 0.05/40 (approximately 0.00125); *P* Values below this threshold were considered statistically significant. Abbreviations: SES, socioeconomic status; WBC, white blood cell; BUN, blood urea nitrogen; eGFR, estimated glomerular filtration rate; LDL, low-density lipoprotein; HDL, high-density lipoprotein; AST, aspartate aminotransferase; ALT, alanine aminotransferase; PCSK9, proprotein convertase subtilisin kexin9

Various statistical approaches have been used to analyze the association between Lp(a) level variability, baseline Lp(a) level risk categories, and risk reclassification. Descriptive statistics were calculated to summarize the distribution of the Lp(a) level variability across the baseline Lp(a) level risk categories. Logistic regression analysis was conducted to explore the predictors of Lp(a) level variability, including baseline and follow-up Lp(a) level risks. The results are presented as odds ratios (ORs) with 95% confidence intervals (CIs). A mixed-effects model was used to account for potential random effects associated with the follow-up Lp(a) level risk.

Univariate and multivariate analyses were conducted to identify the predictors of high Lp(a) level variability. Three different models were used: a multivariate model including all significant variables from the univariate analysis, a stepwise selection model that iteratively chose the best variable combination, and a least absolute shrinkage and selection operation (LASSO)-based model to minimize overfitting. Comparing these models allowed for cross-validation of the findings and ensured robustness by identifying consistent predictors across different analytical approaches. Predictor variables considered included demographic factors, clinical history, laboratory values, and treatment history, specifically: age, sex, alcohol use, smoking status, body mass index (BMI), socioeconomic status, initial and follow-up Lp(a) levels (per 10 mg/dL increment), recent hospital admission, prior cardiovascular events (myocardial infarction, stroke, heart failure), comorbidities (hypertension, diabetes mellitus, dyslipidemia, chronic kidney disease, liver disease, thyroid disease), hemoglobin level (per 1 g/dL decrease), white blood cell (WBC) count (per 1,000 cells/µL increase), platelet count (per 10,000 cells/µL increase), eGFR (per 5 mL/min/1.73 m² decrease), lipid profile and glucose levels (total cholesterol, LDL-cholesterol, HDL-cholesterol, triglycerides, glucose per 10 mg/dL increment), liver enzyme levels (aspartate aminotransferase, alanine aminotransferase per 10 mg/dL increment), blood urea nitrogen, creatinine, and total bilirubin levels (per 1 mg/dL increment), albumin levels (per 1 mg/dL decrement), and treatments (antiplatelet medications, antihypertensive medications, antidiabetic medications, statins, ezetimibe, and PCSK9 inhibitors). Statistical significance was set at *P* < 0.05, and all analyses were conducted using R version 4.1.2.

### Ethical considerations

This study was approved by the Institutional Review Boards of the participating hospitals. The requirement for written informed consent was waived because of the retrospective nature of the study and the use of anonymized data.

## Results

### Correlation between baseline Lp(a) levels and changes in Lp(a) levels

**Fig. 1:**
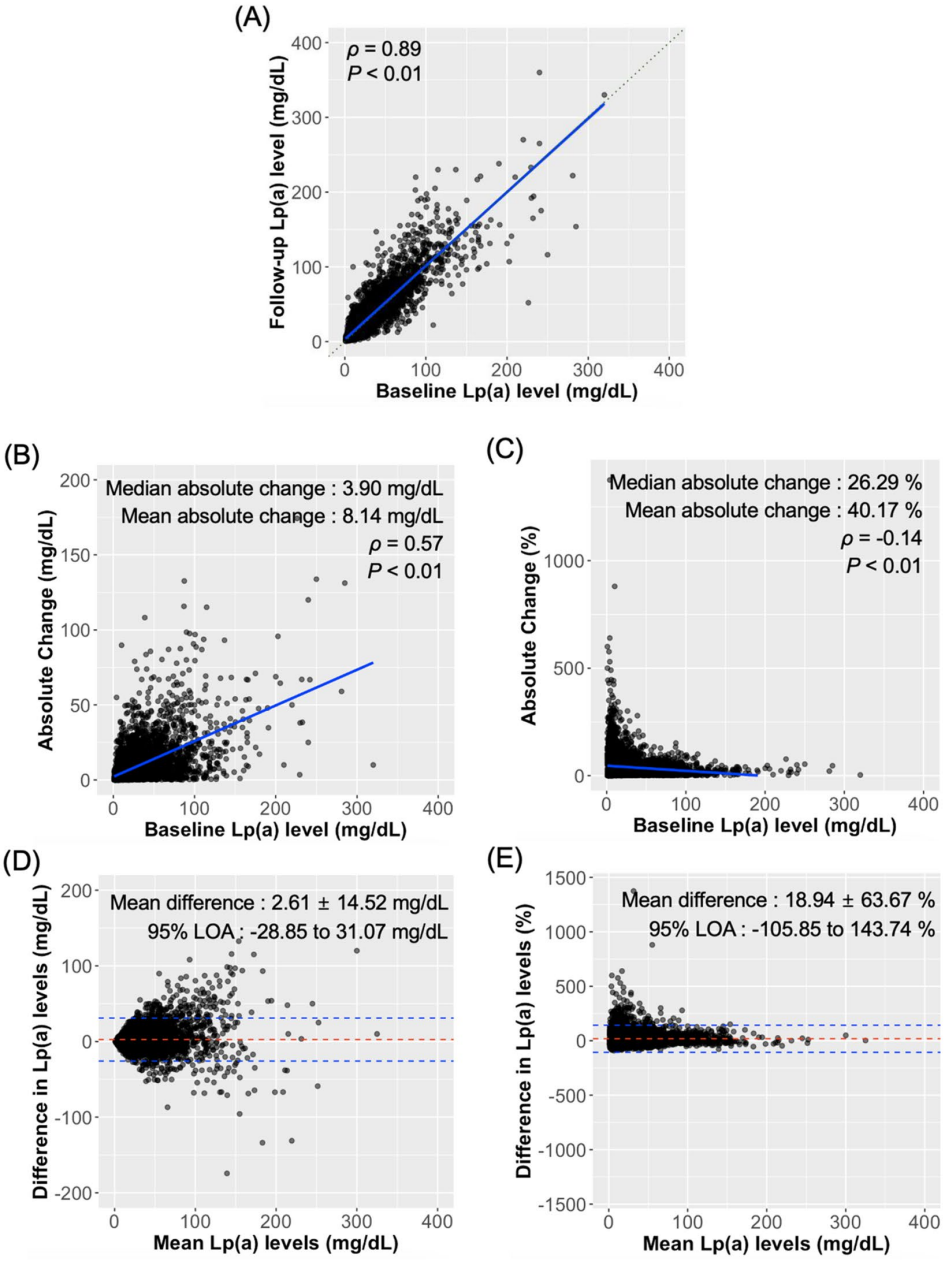
Relationship between baseline Lp(a) levels and changes in Lp(a) levels. (**A**) Correlation between baseline and follow-up Lp(a) levels. (**B**) Absolute change in Lp(a) levels (mg/dL) by baseline Lp(a) level. (**C**) Percentage change in Lp(a) levels compared to baseline Lp(a) levels. (**D**) Bland–Altman plot for the absolute change in Lp(a) levels. (**E**) Bland–Altman plot for the percentage change in Lp(a) levels. The solid blue lines in panels (**A**-**C**) indicate regression slopes. In panels (**D**, **E**), the dashed red line represents the mean difference and the dashed blue lines denote the 95% limits of agreement (LOA). Lp(a), lipoprotein(a)

The mean follow-up duration was 442.1 ± 330.1 days with a median of 361 days. A strong positive correlation was observed between the baseline and follow-up Lp(a) levels (Fig. 1(A); ρ = 0.89, *P* < 0.01), indicating overall consistency in the measurements across the study period. However, substantial individual variability was observed. Figure 1(B) demonstrates a moderate positive correlation (ρ = 0.57) between baseline Lp(a) levels and the absolute change in Lp(a) levels (mg/dL), with a median absolute change of 3.9 mg/dL (range, 0.0–174.3 mg/dL). In contrast, Fig. 1(C) reveals a weak negative correlation (*r* = − 0.132) between baseline Lp(a) levels and percentage change, with a median percentage change of 26.3% (range, 0.0–1,375.0%). The Bland–Altman plot for absolute differences (Fig. 1D) showed a mean difference (bias) of 2.61 mg/dL with 95% limits of agreement from − 28.85 to 31.07 mg/dL, whereas the plot for percentage differences (Fig. 1E) indicated a mean difference of 18.94%, with 95% limits of agreement ranging from − 107.36 to 143.74%. These results suggest that despite a strong overall correlation, individual Lp(a) level changes exhibited considerable variability, particularly when expressed as percentages, highlighting the complex dynamics of Lp(a) level fluctuations.

To address the potential misclassification of variability, particularly in patients with low baseline Lp(a) levels, “high Lp(a) variability” was defined using a combined criterion that required both an absolute change of > 10 mg/dL and a relative change of > 25%. This approach captures clinically significant fluctuations in Lp(a) levels more robustly, thereby mitigating the risk of overestimating variability based solely on minor absolute changes in patients with low baseline levels.

In comparative analyses using LDL-cholesterol as a reference biomarker, Lp(a) levels showed significantly smaller median absolute (0.7 mg/dL [interquartile range {IQR} 8.4] vs. -12 mg/dL [IQR 51.0], *P* < 0.01) and relative variability (6.0% [IQR 56.8%] vs. -14.2% [IQR 49.7%], *P* < 0.01). Baseline LDL-cholesterol levels were strongly inversely correlated with LDL-cholesterol level variability (rho = -0.61 for absolute, rho = -0.54 for relative changes, both *P* < 0.01), whereas baseline Lp(a) levels showed minimal or negligible correlation with subsequent variability (rho = -0.01 absolute, *P* = 0.45; rho = -0.12 relative, *P* < 0.01) (Supplementary Table S1).

### Clinical characteristics by Lp(a) level variability

Among the 5,305 patients, 1,057 (19.9%) exhibited high Lp(a) level variability, whereas 4,248 (80.1%) exhibited low variability (Table 1). After adjusting for multiple comparisons using a Bonferroni correction, patients in the high-variability group were significantly older than those in the low variability group (65.2 ± 13.1 vs. 62.5 ± 14.7 years, respectively, *P* < 0.001). In contrast, sex distribution, smoking status, alcohol consumption habits, low socioeconomic status, and BMI did not differ significantly between the groups. Notably, the prevalence of hypertension (84.3% vs. 74.9%), diabetes mellitus (54.2% vs. 45.6%), dyslipidemia (90.7% vs. 83.9%), and prior stroke (32.5% vs. 25.1%) was significantly higher in the high-variability group (all *P* < 0.001). Regarding laboratory parameters, high-variability was associated with statistically lower hemoglobin levels (13.2 ± 1.0 vs. 13.5 ± 1.7 g/dL, *P* < 0.001) and elevated WBC counts (7.2 ± 2.7 vs. 6.7 ± 2.3 × 10^3^/µL, *P* < 0.001). Additionally, serum glucose levels were significantly higher in the high-variability group (123.5 ± 47.6 vs. 114.9 ± 37.4 mg/dL, *P* < 0.001). Notably, both baseline and follow-up Lp(a) levels were significantly elevated in patients with high-variability (*P* < 0.001 for both comparisons). The high-variability group had significantly greater use of antiplatelet medications, antihypertensive agents, antidiabetic medications, and statins (all *P* < 0.001), whereas differences in ezetimibe (*P* = 0.002) and PCSK9 inhibitor use (*P* = 1.00) were not statistically significant. These findings suggest that after rigorous adjustment for multiple comparisons, high Lp(a) level variability is associated with older age, a greater burden of cardiovascular comorbidities, and increased use of cardiovascular medications, reflecting a more adverse cardiovascular risk profile.

### Risk reclassification of Lp(a) levels

**Fig. 2:**
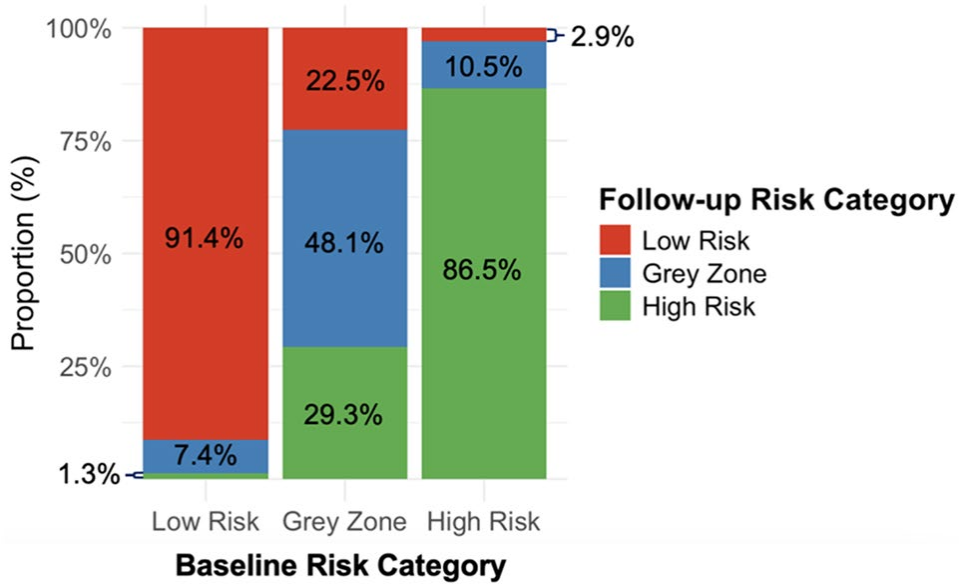
Risk reclassification of Lp(a) levels based on follow-up measurements. Percentages within each bar represent the proportion of patients within each baseline category who remained in the same risk category or shifted to a different risk category at follow-up. Lp(a), lipoprotein(a)

Baseline and follow-up Lp(a) levels were stratified into three risk categories: low-risk (< 30 mg/dL), gray-zone (30–49 mg/dL), and high-risk (≥ 50 mg/dL). Reclassification analysis showed that 91.4% of patients initially classified as low-risk remained in the low-risk category at follow-up, 7.4% moved to the gray-zone, and 1.3% transitioned to high-risk (Table 2; Fig. 2). Among those initially in the gray-zone, 48.1% remained, 22.5% changed to low-risk, and 29.3% shifted to high-risk. For patients classified as high-risk at baseline, 86.5% remained in the high-risk category, 10.5% moved to the gray-zone, and 2.9% to the low-risk category. These findings indicate that the Lp(a) risk classification remained largely stable over time in the low- and high-risk categories, whereas patients in the gray-zone exhibited greater variability, with approximately half (51.9%) being reclassified into a different risk category.

**Table 2:** Risk reclassification with Lp(a) variability

Baseline Lp(a) Risk	Follow-up Lp(a) Risk	Lp(a) variability	*N* (%)
Low Risk Low Lp(a) Variaility 3470 (87.5%) High Lp(a) Variability 497 (12.5%)	Low Risk 3624 (91.4%)	Low Variability	3386 (93.4%)
		High Variability	238 (6.6%)
	Grey Zone 292 (7.4%)	Low Variability	84 (28.8%)
		High Variability	208 (71.2%)
	High Risk 51 (1.3%)	High Variability	51(100%)
Grey Zone Low Lp(a) Variability 345 (55.9%) High Lp(a) Variaility 272 (44.1%)	Low Risk 139 (22.5%)	Low Variability	64 (46.0%)
		High Variability	75 (54.0%)
	Grey Zone 297 (48.1%)	Low Variability	256 (86.2%)
		High Variability	41 (13.8%)
	High Risk 181 (29.3%)	Low Variability	25 (13.8%)
		High Variability	156 (86.2%)
High Risk Low Lp(a) Variability 433 (60.1%) High Lp(a) Variaility 288 (39.9%)	Low Risk 21 (2.9%)	High Variability	21 (100%)
	Grey Zone 76 (10.5%)	Low Variability	25 (32.9%)
		High Variability	51 (67.1%)
	High Risk 624 (86.5%)	Low Variability	408 (65.4%)
		High Variability	216 (34.6%)

The table presents the distribution of patients by baseline Lp(a) risk category, follow-up Lp(a) risk category, and Lp(a) variability. Counts and percentages are shown for each combination to illustrate the dynamics of risk reclassification in relation to Lp(a) variability

### Association of Lp(a) level variability with baseline risk and risk reclassification

High Lp(a) level variability was observed in 12.5% of the “low-risk” group, 44.1% of the “gray-zone” group, and 39.9% of the “high-risk” group, suggesting a trend toward increased variability with higher baseline risk. Logistic regression analysis was conducted to evaluate the relationship between Lp(a) level variability and the risk categories derived from the baseline and follow-up measurements. Compared with patients in the low-risk category, those classified as high-risk at baseline had significantly lower odds of exhibiting low Lp(a) level variability (OR = 0.36, 95% CI = 0.26–0.50, *P* < 0.01). Moreover, patients with ”gray-zone” and high-risk classifications at follow-up were associated with markedly increased odds of high Lp(a) level variability, with ORs of 9.60 (95% CI = 7.63–12.09, *P* < 0.01) and 21.44 (95% CI = 15.58–29.71, *P* < 0.01), respectively.

The mixed-effects model further confirmed these findings, accounting for random effects related to follow-up Lp(a) level risk (Akaike information criterion [AIC] = 4,335.5, *P* < 0.05). Patients in the “high-risk” baseline category were significantly more likely to exhibit high Lp(a) variability, even after accounting for individual-level variability. Overall, the analyses indicated a significant relationship between baseline Lp(a) risk categories, Lp(a) variability, and risk reclassification, emphasizing the importance of monitoring Lp(a) levels for effective cardiovascular risk stratification.

### Factors influencing high Lp(a) level variability

A comprehensive analysis of the 40 variables using univariate logistic regression revealed that many factors were significantly associated with high Lp(a) level variability (*P* < 0.05) (Table 3). A multivariate logistic regression model, constructed using variables with *P* < 0.1 in the univariate analysis, included 30 variables. Key associations in this model were observed for baseline Lp(a) levels (OR = 0.67, 95% CI = 0.62–0.71, *P* < 0.01), follow-up Lp(a) levels (OR = 1.85, 95% CI = 1.74–1.97, *P* < 0.01), BMI (OR = 0.97, 95% CI = 0.95-1.00, *P* = 0.02), hemoglobin levels (OR = 1.08, 95% CI = 1.03–1.14, *P* < 0.01), platelet counts (OR = 1.01, 95% CI = 1.00-1.02, *P* = 0.01), and serum glucose levels (OR = 1.03, 95% CI = 1.01–1.05, *P* < 0.01).

**Table 3:** Logistic regression analysis of factors associated with high Lp(a) variability

	Univariable		Multivariable (*p* ≤ 0.1)		Multivariable (Stepwise)		Multivariable (LASSO)	
	OR (95% CI)	*P* Value	OR (95% CI)	*P* Value	OR (95% CI)	*P* Value	OR (95% CI)	*P* Value
Age	**1.01(1.01–1.02)**	**< 0.01**	1.00(1.00-1.01)	0.28			1.00(1.00-1.01)	0.28
Male	1.03(0.90–1.19)	0.66					1.12(0.93–1.35)	0.24
Smoking	1.04(0.91–1.19)	0.59					1.04(0.86–1.26)	0.70
Drinking	1.00(0.87–1.15)	0.96					0.91(0.74–1.10)	0.33
Low SES	**1.28(1.03–1.60)**	**0.03**	1.07(0.83–1.38)	0.62			1.07(0.83–1.38)	0.62
**Body mass index**	**0.98(0.96-1.00)**	**0.04**	**0.97(0.95-1.00)**	**0.02**	**0.97(0.95–0.99)**	**0.01**	**0.98(0.95-1.00)**	**0.03**
Hypertension	**1.77(1.48–2.13)**	**< 0.01**	1.16(0.86–1.56)	0.34			1.15(0.86–1.56)	0.35
Diabetes mellitus	**1.41(1.23–1.62)**	**< 0.01**	1.11(0.88–1.39)	0.39			1.12(0.89–1.41)	0.33
Dyslipidemia	**1.84(1.46–2.30)**	**< 0.01**	0.97(0.70–1.34)	0.84			0.94(0.68–1.31)	0.72
Chronic kidney disease	**1.51(1.23–1.85)**	**< 0.01**	1.23 (0.83–1.82)	0.31			1.25(0.84–1.85)	0.28
Liver Disease	1.13(0.93–1.37)	0.22					1.21(0.96–1.54)	0.11
Thyroid Disease	1.11 (0.91–1.37)	0.30					1.09(0.84–1.40)	0.52
Prior myocardial infarction	1.23(0.95–1.61)	0.12					0.81(0.58–1.15)	0.25
Prior stroke	**1.42(1.22–1.64)**	**< 0.01**	1.07(0.88–1.30)	0.50			1.06(0.87–1.28)	0.59
Prior heart failure	**1.37(1.04–1.79)**	**0.02**	1.14(0.83–1.57)	0.41			1.16(0.84–1.60)	0.36
**Hemoglobin**	**1.11(1.07–1.16)**	**< 0.01**	**1.08(1.03–1.14)**	**< 0.01**	**1.09(1.04–1.14)**	**< 0.01**	**1.10(1.04–1.17)**	**< 0.01**
**WBC count**	**1.08(1.05–1.11)**	**< 0.01**	1.03(1.00-1.06)	0.10	**1.04(1.00-1.07)**	**0.03**	1.03(0.99–1.06)	0.11
**Platelet count**	**1.01(1.00-1.02)**	**0.05**	**1.01(1.00-1.02)**	**0.01**	**1.01(1.00-1.02)**	**0.02**	**1.01(1.00-1.03)**	**< 0.01**
BUN	**1.02(1.01–1.03)**	**< 0.01**	1.00(0.99–1.02)	0.49			1.00(0.99–1.02)	0.50
Creatinine	**1.09(1.01–1.16)**	**0.02**	1.04(0.92–1.18)	0.48			1.04 (0.92–1.18)	0.54
- eGFR	**1.03(1.02–1.05)**	**< 0.01**	0.97(0.93–1.01)	0.15			0.97(0.93–1.01)	0.13
Total cholesterol	1.01(0.99–1.02)	0.37					1.01(0.96–1.05)	0.77
LDL-cholesterol	1.01(0.99–1.03)	0.28					1.00(0.96–1.06)	0.85
**HDL-cholesterol**	**0.90(0.86–0.95)**	**< 0.01**	0.94(0.88–1.01)	0.08	**0.94(0.88-1.00)**	**0.05**	0.94(0.87–1.02)	0.13
Triglyceride	**1.01(1.00-1.02)**	**< 0.01**	1.01(1.00-1.01)	0.09	1.01(1.00-1.01)	0.11	1.01(1.00-1.02)	0.27
**Glucose**	**1.05(1.03–1.06)**	**< 0.01**	**1.03(1.01–1.05)**	**< 0.01**	**1.03(1.01–1.05)**	**< 0.01**	**1.03(1.01–1.05)**	**< 0.01**
AST	1.01(1.00-1.03)	0.10	1.00(0.99–1.02)	0.69			1.00(0.98–1.03)	0.66
ALT	1.00(0.98–1.03)	0.84					1.00(0.96–1.03)	0.83
Total bilirubin	1.07(0.89–1.28)	0.49					1.15(0.92–1.43)	0.22
Albumin	**1.58(1.35–1.85)**	**< 0.01**	1.15(0.92–1.43)	0.22	1.17(0.96–1.44)	0.13	1.18(0.94–1.47)	0.15
**Baseline Lp(a)**	**1.21(1.18–1.24)**	**< 0.01**	**0.67(0.62–0.71)**	**< 0.01**	**0.67(0.63–0.71)**	**< 0.01**	**0.67(0.63–0.71)**	**< 0.01**
- measured during hospitalization	0.86(0.73–1.02)	0.08	0.98(0.79–1.21)	0.85			1.01(0.82–1.25)	0.92
**Follow-up Lp(a)**	**1.31(1.28–1.34)**	**< 0.01**	**1.85(1.74–1.97)**	**< 0.01**	**1.85(1.74–1.97)**	**< 0.01**	**1.85(1.74–1.97)**	**< 0.01**
- measured during hospitalization	**0.78(0.65–0.94)**	**< 0.01**	0.94(0.74–1.20)	0.61			0.95(0.74–1.21)	0.67
Antiplatelet drug	**1.44(1.25–1.66)**	**< 0.01**	0.99(0.81–1.20)	0.90			1.02(0.84–1.25)	0.84
**Antihypertensive drug**	**1.70(1.46–1.99)**	**< 0.01**	1.25(0.97–1.62)	0.09	**1.43(1.20–1.70)**	**< 0.01**	1.24(0.96–1.61)	0.10
Antidiabetic drug	**1.36(1.18–1.56)**	**< 0.01**	0.91(0.72–1.14)	0.41			0.91(0.71–1.14)	0.41
Statin	**1.58(1.35–1.86)**	**< 0.01**	1.08(0.83–1.42)	0.56			1.09(0.83–1.43)	0.54
Ezetimibe	**1.35(1.17–1.56)**	**< 0.01**	0.93(0.77–1.13)	0.47			0.93(0.77–1.13)	0.48
PCSK9 inhibitor	**2.23(1.18–4.21)**	**0.01**	1.72(0.79–3.78)	0.17			1.83(0.83–4.10)	0.13

Odds ratios (ORs), 95% confidence intervals (CIs), and *P* Values are provided to summarize the associations

Stepwise selection refined the model to 11 variables, retaining similar key predictors, such as baseline and follow-up Lp(a) levels, BMI, hemoglobin levels, platelet counts, and serum glucose levels. This model also highlighted the importance of WBC count (OR = 1.04, 95% CI = 1.00-1.07, *P* = 0.03) and antihypertensive medication use (OR = 1.43, 95% CI = 1.20–1.70, *P* < 0.01).

The LASSO-selected model, encompassing 40 variables, consistently identified the importance of baseline and follow-up Lp(a) levels, BMI, hemoglobin levels, platelet counts, and serum glucose levels.

Model performance was compared using AIC and pseudo R-squared values. The stepwise model demonstrated the best fit, with the lowest AIC value (4,226.3). The pseudo R-squared values were comparable across the LASSO-selected (0.19), full (0.19), and stepwise (0.18) models, indicating similar explanatory power for Lp(a) level variability.

**Fig. 3:**
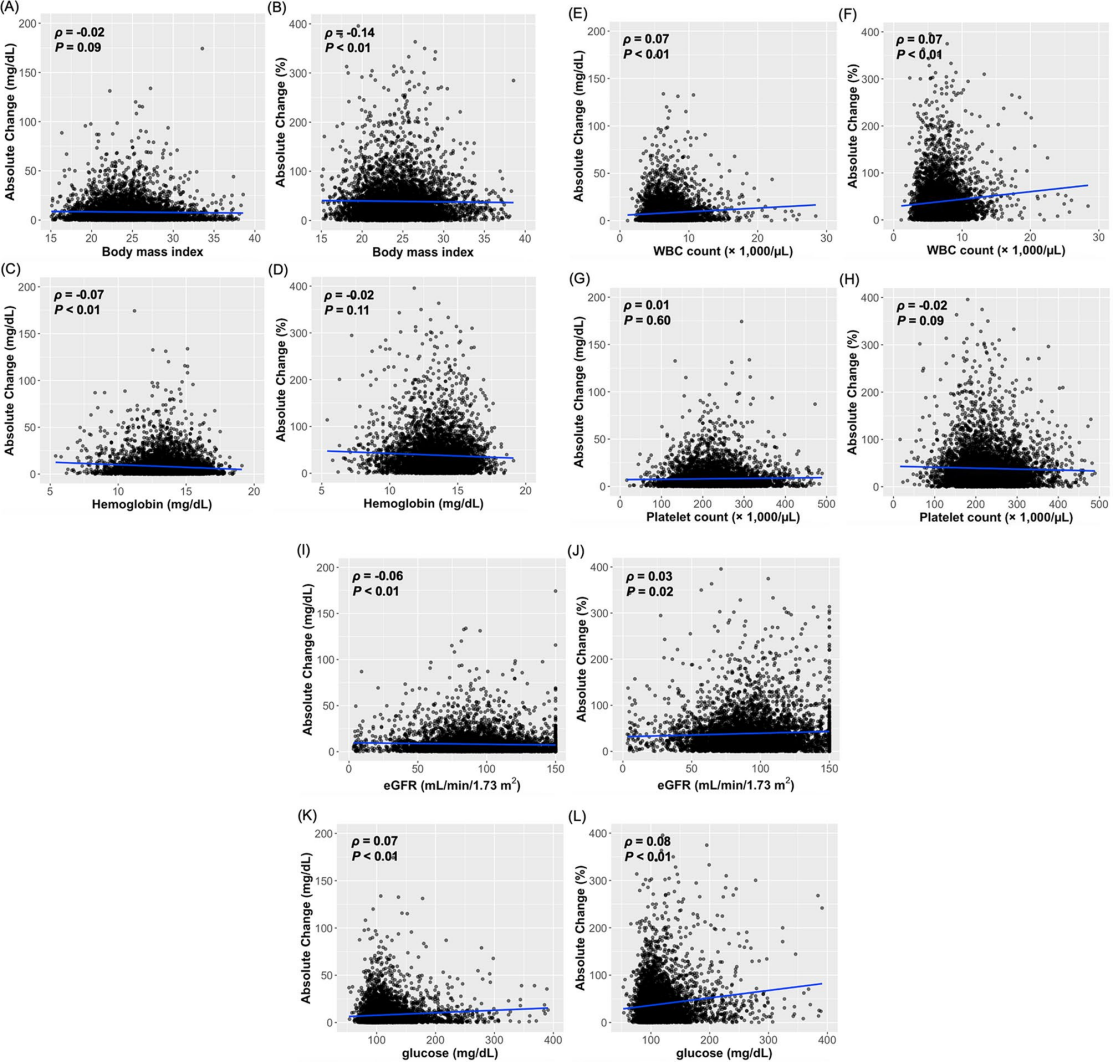
Relationship between key predictors of Lp(a) level variability and changes in Lp(a) levels. Scatter plots illustrate the relationship between the key predictors of Lp(a) level variability and changes in the Lp(a) levels. The left panels (**A**, **C**, **E**, **G**, **I**, and **K**) represent the correlations between key predictors and absolute numeric changes in Lp(a) levels (mg/dL). The right panels (**B**, **D**, **F**, **H**, **J**, and **L**) represent the correlations between key predictors and the absolute values of relative percentage changes (%) in the Lp(a) levels, indicating the magnitude of variability independent of the direction (positive or negative). The blue regression lines indicate linear trends. Lp(a), lipoprotein(a); WBC, white blood cell

To further characterize the relationship between key clinical factors (BMI, hemoglobin level, WBC count, platelet count, eGFR, and serum glucose level) and Lp(a) level variability, Spearman’s rank correlation analysis was performed (Fig. 3). Consistent with the findings from the logistic regression analysis, BMI demonstrated a weak but significant positive correlation with the absolute percentage change in Lp(a) levels (ρ = 0.14, *P* < 0.01), whereas no significant association was observed with absolute numeric changes (ρ = -0.02, *P* = 0.09). Similarly, hemoglobin levels exhibited a weak negative correlation with absolute numeric changes (ρ = -0.07, *P* < 0.01), although no significant association was found with absolute percentage changes (ρ = -0.02, *P* = 0.11). Platelet counts did not demonstrate a meaningful correlation with either absolute numeric (ρ = 0.01, *P* = 0.60) or percentage changes in Lp(a) levels (ρ = -0.02, *P* = 0.09), suggesting a minimal influence on Lp(a) level variability. The eGFR showed a weak but statistically significant inverse correlation with absolute numeric changes (ρ = -0.06, *P* < 0.01) and a weak positive correlation with absolute percentage changes (ρ = 0.03, *P* = 0.02). WBC count was positively correlated with both absolute numeric (ρ = 0.07, *P* < 0.01) and percentage changes in Lp(a) levels (ρ = 0.07, *P* < 0.01). Similarly, glucose levels were positively correlated with both absolute numeric (ρ = 0.07, *P* < 0.01) and percentage changes in Lp(a) levels (ρ = 0.08, *P* < 0.01), further supporting its role as a contributing factor in Lp(a) level variability, as previously indicated by logistic regression analysis. Overall, these findings suggest that, whereas WBC count, eGFR, and glucose level may have modest influences on Lp(a) level variability, platelet count does not appear to be a major contributor.

### Sensitivity and interaction analyses for medication initiation and hospitalization

Sensitivity analyses were performed by separately excluding patients who were taking lipid-lowering medications (statins, ezetimibe, and PCSK9 inhibitors) or were hospitalized for baseline or follow-up Lp(a) level measurements. These sensitivity analyses consistently confirmed the significant associations of baseline (OR range: 0.63–0.73; all *P* < 0.01) and follow-up (OR range: 1.76–1.92; all *P* < 0.01) Lp(a) levels with high Lp(a) variability (Supplementary Table S2). Although some clinical variables, such as BMI, hemoglobin level, platelet count, and glucose level, showed slight variations in statistical significance across the analyses, the overall magnitude and direction of their associations remained stable. Interaction analysis revealed statistically significant interactions only between HDL-cholesterol levels and ezetimibe use (OR = 1.16, 95% CI = 1.00–1.35; *P* = 0.04), and between HDL-cholesterol levels and hospitalization at follow-up (OR = 1.29, 95% CI = 1.07–1.55; *P* < 0.01). No other interaction terms involving BMI, hemoglobin, WBC and platelet counts, glucose, or antihypertensive medication use reached statistical significance, indicating stable associations with Lp(a) level variability independent of medication initiation or hospitalization status.

## Discussion

**Fig. 4:**
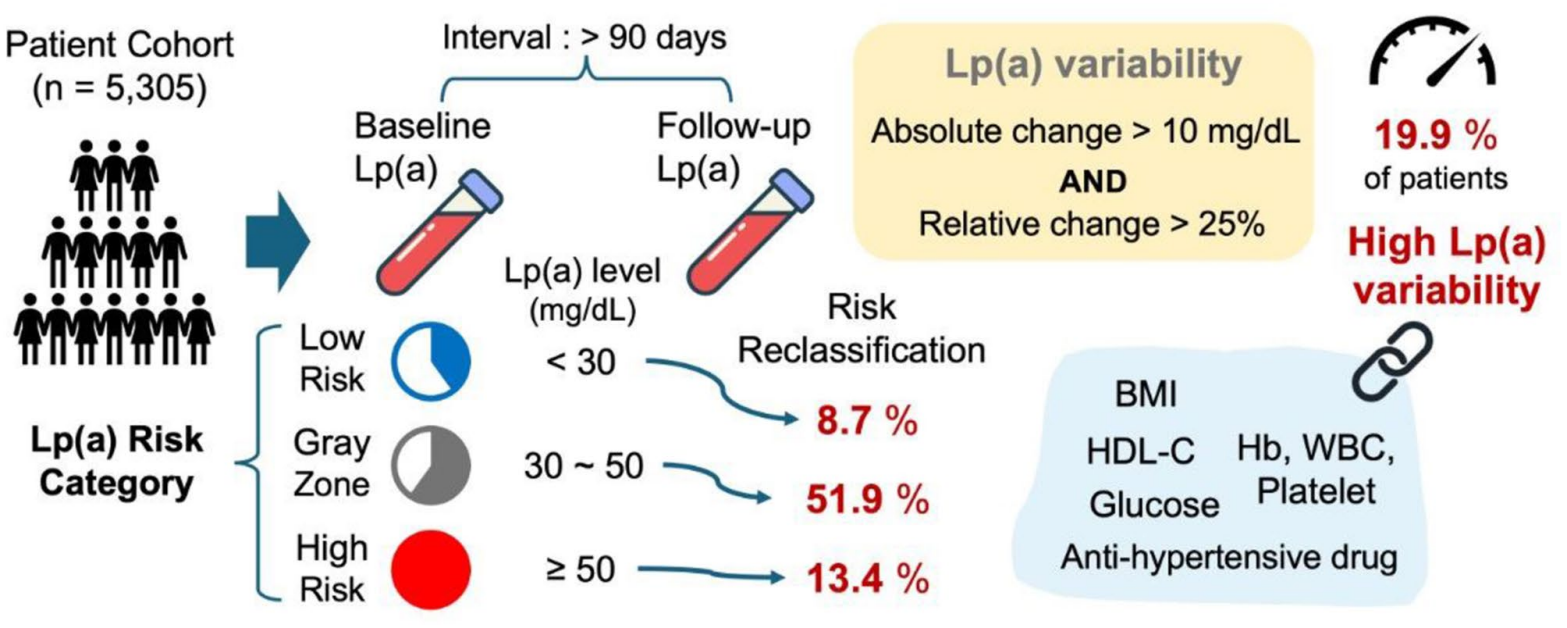
Workflow and key findings of intraindividual Lp(a) level variability in cardiovascular risk stratification. This diagram illustrates the workflow and key findings of the study. The process begins with a cohort of 5,305 patients undergoing baseline and follow-up Lp(a) level measurements (≥ 90 days apart). Lp(a) variability was calculated using the following dual criteria: absolute change greater than 10 mg/dL and relative change > 25%, with high-variability observed in 19.9% of patients. Subsequent risk reclassification analysis indicated that nearly 51.9% of the patients were in the intermediate ‘gray-zone’ shift categories over time, underscoring the potential of serial Lp(a) assessments to enhance cardiovascular risk stratification. Key clinical predictors, such as BMI, hemoglobin level, WBC count, platelet count, serum glucose level, HDL-cholesterol level, and use of antihypertensive medications, further support the association between Lp(a) level variability and adverse cardiovascular profiles. Lp(a), lipoprotein(a); BMI, body mass index; WBC, white blood cell; HDL, high-density lipoprotein

This study demonstrated that Lp(a) levels exhibited substantial intraindividual variability, with approximately 19.9% of the patients classified as having high-variability. Although baseline and follow-up Lp(a) level measurements were strongly correlated (*r* = 0.89, *P* < 0.01), the absolute change in Lp(a) levels showed only a moderate association with baseline levels (ρ = 0.57), whereas the percentage change exhibited a weak negative correlation (*r* = − 0.132). Notably, high Lp(a) level variability was significantly associated with adverse cardiovascular risk profiles, including a higher prevalence of hypertension, diabetes mellitus, dyslipidemia, and prior stroke, as well as unfavorable laboratory parameters such as lower hemoglobin levels, elevated WBC counts, and increased serum glucose levels. Additionally, risk reclassification analysis revealed that whereas Lp(a) level risk categories remain relatively stable among patients at the extremes (low- and high-risk), those in the intermediate “gray-zone” experience considerable reclassification over time. These findings provide novel insights into the dynamic nature of Lp(a) level fluctuations and their clinical characteristics, underscoring the need for future prospective studies to determine the potential utility of serial Lp(a) level measurements to refine cardiovascular risk stratification and guide personalized treatment strategies (Fig. 4).

The definition of Lp(a) level variability differed between the studies, which impacted the interpretation and comparison of the results. Previous studies used single criteria, such as the UK Biobank study, which defined variability as a change of ≥ 25 nmol/L (approximately 10 mg/dL), or the IONIS-APO(a)Rx and IONIS-APO(a)-LRx clinical trials, which used a > 25% change [11, 12]. In contrast, in this study, high Lp(a) level variability was defined as an absolute change of > 10 mg/dL and a relative change of > 25%. This dual definition allows for a more comprehensive assessment of variability across the spectrum of Lp(a) levels. This approach addresses the limitations of using only one criterion because relative changes can be misleading for low initial values, whereas absolute changes may not capture clinically significant variations at higher levels.

Indeed, the present study demonstrated a moderate positive correlation (*ρ* = 0.57) between baseline Lp(a) levels and absolute changes, and a weak negative correlation (*ρ* = -0.14) between baseline Lp(a) levels and percentage changes. This suggests that individuals with higher baseline levels might have more stable Lp(a) production or clearance rates relative to their baseline levels, despite experiencing larger absolute fluctuations. This relationship between absolute and relative changes highlights the inadequacy of using a single metric to define clinically significant Lp(a) level variability. Therefore, a more nuanced approach that considers both absolute and relative changes is necessary for an accurate risk stratification. The comprehensive definition of variability combined with risk category transition analysis provided a more detailed understanding of the Lp(a) level dynamics and its potential impact on cardiovascular risk assessment.

To further clarify the clinical relevance of Lp(a) level variability, a direct comparison with LDL-cholesterol levels, an established lipid marker known for substantial temporal fluctuations, was performed. The findings showed that both absolute and relative variabilities were significantly lower for Lp(a) levels than for LDL-cholesterol levels (*P* < 0.01). Additionally, baseline LDL-cholesterol levels strongly predicted subsequent LDL-cholesterol level fluctuations, whereas Lp(a) level variability showed only minimal dependence on baseline Lp(a) levels. These comparative analyses indicate that Lp(a) levels are relatively more stable over time than LDL-cholesterol levels, emphasizing their potential as a reliable biomarker for assessing long-term cardiovascular risk.

The stability of the Lp(a) level risk classifications observed in this study, particularly for high- and low-risk groups (86.5% and 91.4%, respectively, remaining in their categories), is consistent with the findings of previous studies (89.9% and 96.4%, respectively [8] and 93% and 92%, respectively [4]). This consistency across different populations and study designs reinforces the reliability of a single Lp(a) level measurement for individuals in these risk categories. This suggests that a single Lp(a) level measurement may be sufficient for the initial risk stratification in these groups. This approach may lead to more efficient and cost-effective screening protocols. Greater variability was also observed in the intermediate-risk “gray-zone” category, with 51.9% changing categories compared to 51.2% [8] and 53% [4] in previous studies. This consistency highlights a critical aspect of Lp(a) level risk assessment; individuals with intermediate Lp(a) levels may require more frequent monitoring or additional risk stratification.

The examination of Lp(a) level variability over a shorter median follow-up period of 1.07 years in this study provides valuable insights into short-term fluctuations in Lp(a) levels, complementing findings from studies with longer follow-up periods [8, 13]. This relatively short interval allows for a more focused assessment of Lp(a) level fluctuations that may occur over a clinically relevant timeframe. Compared with the results reported by Awad et al., [8] which had a median follow-up of 4.5 years, the present study revealed a similar proportion (53%) of individuals in the intermediate “gray-zone” transitioning to other risk categories, consistent with the 51.2% observed previously. This suggests that the reliability of the Lp(a) level variability assessment over a ~ 1-year interval appears to be sufficient for clinical decision-making, particularly for individuals in the intermediate-risk category. However, it is important to note that the optimal testing interval may vary depending on the individual risk factors and baseline Lp(a) levels.

Multiple statistical models have identified several significant predictors of high Lp(a) level variability. Consistent across models, baseline and follow-up Lp(a) levels were strong predictors. Additionally, hematological parameters (hemoglobin levels, WBC counts, and platelet counts) and metabolic factors (BMIs, serum glucose levels, and HDL-cholesterol levels) were found to be significant. These findings offer insights into the complex interplay between the factors influencing Lp(a) level variability.

The findings of the present study differ from those reported by Awad et al., who found associations between intraindividual Lp(a) changes of ≥ 10 mg/dL and female sex, a history of atherosclerotic cardiovascular disease, statin therapy, and elevated LDL-cholesterol levels (≥ 100 mg/dL) [8]. This discrepancy may be attributed to differences in the study populations, analytical approaches, or the use of a more stringent definition of high Lp(a) variability, which required an absolute change of > 10 mg/dL and a relative change of > 25%.

The association between WBC count and high Lp(a) level variability aligns with the results of previous studies, suggesting a link between Lp(a) levels and inflammatory processes. Lp(a) promotes monocyte extravasation, endothelial cell activation, and the upregulation of adhesion molecules such as ICAM-1 [14]. Oxidized phospholipids carried by Lp(a) can trigger inflammatory responses and activate MAPK and NF-κB pathways in monocytes, leading to increased inflammatory gene expression [15]. Zheng et al. reported a positive correlation between Lp(a) level variability and the mean follow-up C-reactive protein levels in patients who underwent percutaneous coronary intervention, further supporting this connection [16]. 

The identification of serum glucose levels and BMI as predictors suggests a potential relationship between metabolic factors and Lp(a) level variability. This finding highlights the importance of considering metabolic health in the context of Lp(a) dynamics. Furthermore, the inclusion of eGFRs in the model highlights the potential role of renal function in influencing Lp(a) metabolism and variability.

Although measuring inflammatory markers such as high-sensitivity C-reactive protein (hs-CRP) could provide deeper insights into the inflammatory basis of Lp(a) level variability, substantial missing data for hs-CRP levels (~ 47.5%) limited the reliable statistical adjustment in this study. This is an important limitation, and further research incorporating comprehensive inflammatory biomarker data is required to elucidate these relationships more precisely.

Although the present study demonstrated an association between elevated Lp(a) levels and several cardiovascular risk factors, the specific genetic and demographic features of the study population may limit the generalizability of these findings to other ethnic groups. Previous research has shown that median Lp(a) levels vary widely among racial and ethnic populations, largely because of differences in genetic factors such as KIV copy numbers and LPA gene polymorphisms [3]. The UK Biobank study also reported median Lp(a) levels ranging from 31 mg/dL in Hispanic individuals to 65 mg/dL in non-Hispanic Black individuals, with similar hazard ratios for cardiovascular events per 50 nmol/L increase in Lp(a) levels across these groups [10]. Given these established disparities, the patterns of Lp(a) level variability and clinical implications observed in the Korean cohort may differ from those in other populations. Therefore, validation of these findings in more diverse cohorts, including various Asian subgroups, as well as African, European, and South Asian populations, is warranted.

Recent studies have demonstrated the dynamic nature of Lp(a) levels in acute cardiovascular conditions. Ziogos et al. showed that Lp(a) levels in individuals with acute myocardial infarctions were significantly higher six months after the initial event [17]. Vavuranakis et al. also observed a significant increase in Lp(a) levels during the acute-phase of acute myocardial infarction [18]. Importantly, this acute-phase elevation was attenuated in patients receiving evolocumab, a PCSK9 inhibitor, highlighting the potential role of PCSK9 inhibition in stabilizing Lp(a) levels.

Recent meta-analyses and clinical trials have shown that PCSK9 inhibitors reduce Lp(a) levels by 27% on average [19]. This effect was attributed to decreased Lp(a) production and enhanced degradation via increased LDL receptor recycling. However, because of the limited number of patients receiving PCSK9 inhibitors (44 patients, 0.8% of the cohort), a robust analysis of the association between PCSK9 inhibitor use and Lp(a) level variability was not feasible in the present study. Although this study provides valuable insights into Lp(a) level variability in a large real-world cohort, it highlights the need for further research to elucidate the long-term effects of PCSK9 inhibitors on Lp(a) level variability.

Additionally, recent studies have emphasized that elevated Lp(a) levels not only increase the risk of premature coronary events but also contribute significantly to the complexity of coronary artery disease. For instance, the RELACS study demonstrated that higher Lp(a) levels correlated with younger age at initial coronary events and greater coronary anatomical complexity, as indicated by elevated SYNTAX and Gensini scores [20]. These findings align with the present study’s results regarding Lp(a) level variability and its association with heightened cardiovascular risk, further supporting the clinical relevance of monitoring and managing elevated Lp(a) levels as part of comprehensive cardiovascular risk stratification and management strategies.

### Study strengths and limitations

This study has several strengths. First, a large multicenter cohort from tertiary university hospitals was analyzed, enabling robust statistical analyses and enhanced generalizability within the context of Korean clinical practice. Second, a comprehensive approach was used to define high Lp(a) level variability using both absolute (> 10 mg/dL) and relative (> 25%) changes to provide a nuanced and clinically relevant assessment. Additionally, the study used advanced statistical methods, including mixed-effects modeling and LASSO regression, to account for potential confounders and enhance the robustness and reliability of the identified predictors. Comparative analyses of LDL-cholesterol levels further emphasized the unique stability characteristics of Lp(a) as a biomarker, providing additional clinical insights.

This study had some limitations. First, as a retrospective observational study, the analysis was inherently subject to selection bias and confounding factors that may not have been fully accounted for despite the use of multivariate regression models. Additionally, repeated Lp(a) level measurements in this cohort may reflect clinical selection or interventions. However, considering the widespread use of routine health checkups and low-cost laboratory tests, including Lp(a) levels, in Korea, the cohort likely represents a combination of routine follow-up and targeted clinical management rather than strictly selected clinical cases. Although a wide range of clinical variables were included, unmeasured confounders, such as genetic factors influencing Lp(a) levels and variability, may have affected the results. Additionally, the measurement of Lp(a) levels was limited to only two time points, potentially underrepresenting the long-term variability of these concentrations. Furthermore, the analysis focused primarily on the magnitude of the changes in Lp(a) levels rather than the direction of these changes (i.e., increases versus decreases), which may offer additional clinical insights. Moreover, the study cohort was derived from tertiary care centers and predominantly comprised high-risk patients with a high frequency of statin use and treatments for hypertension and diabetes mellitus, thereby limiting the generalizability of the findings to the general population. Future studies incorporating serial assessments over extended follow-up periods are warranted to comprehensively elucidate the dynamic fluctuations in Lp(a) levels and their associated clinical implications. Second, although the mass-based measurement of Lp(a) levels used in this study provided reliable data, it may limit its direct comparability with investigations using particle concentration assays. Nonetheless, recent head-to-head comparisons have demonstrated that various Lp(a) level measurement methods yield comparable prognostic performances [21]. Third, the current study did not explicitly account for acute cardiovascular events occurring between the Lp(a) level measurements. Recent research by Ziogos et al. showed that Lp(a) levels in individuals with acute myocardial infarctions are significantly higher six months after the initial event [17]. This suggests that a single measurement of Lp(a) levels in the peri-infarction setting may not accurately predict the risk of Lp(a)-associated coronary artery disease risk in the post-infarction period. Although variables related to Lp(a) levels measured during hospitalization were included, the potential impact of acute events on Lp(a) level variability could not be entirely ruled out. Furthermore, the reclassification scheme based on a fixed Lp(a) level threshold introduces inherent limitations. Minor differences near the cutoff (e.g., 49 mg/dL vs. 51 mg/dL) may result in apparently discordant risk categorizations, potentially oversimplifying the continuous nature of cardiovascular risk associated with Lp(a) levels. Furthermore, the large sample size may have resulted in statistically significant differences in variables with minimal absolute changes (e.g., small variations in hemoglobin levels or BMIs), thereby limiting the clinical or biological relevance of these findings. Consequently, these marginal effect sizes necessitate cautious interpretation when integrated into overall cardiovascular risk assessments. Finally, although the current study primarily examined the associations between Lp(a) level variability and established cardiovascular risk factors, it did not extend to the assessment of clinical outcomes, such as myocardial infarctions, strokes, or mortality. Future studies should incorporate clinical endpoints to ascertain whether Lp(a) level variability can independently predict adverse cardiovascular events and overall mortality.

## Conclusions

This study demonstrated that intraindividual variability in Lp(a) levels was significantly associated with adverse cardiovascular risk factors and dynamic risk reclassification, particularly in patients with intermediate baseline levels. These findings highlight the potential of serial Lp(a) level assessments to refine cardiovascular risk stratification.

## Electronic supplementary material

Below is the link to the electronic supplementary material.

Supplementary Material 1

## Data Availability

No datasets were generated or analysed during the current study.

## Abbreviations

Lp(a): Lipoprotein(a)
OMOP-CDM: Observational medical outcomes partnership common data model
LDL: Low-density lipoprotein
HDL: High-density lipoprotein
eGFR: Estimated glomerular filtration rate
LASSO: Least absolute shrinkage and selection operator
OR: Odds ratio
CI: Confidence interval
IQR: Interquartile range
BMI: Body mass index
WBC: White blood cell
PCSK9: Proprotein convertase subtilisin/kexin type 9
AIC: Akaike information criterion
