# Supplementary material for: Predictors of lipoprotein(a) variability in clinical practice and their impact on cardiovascular risk
Source: Lipids Health Dis. 2025 Jul 23;24:250. doi: 10.1186/s12944-025-02666-8 (PMC12288285; doi:10.1186/s12944-025-02666-8)
Supplement: Supplementary file 1 — Supplementary Material 1 [file 12944_2025_2666_MOESM1_ESM.docx]

**Supplementary table S1. Comparison of variability between Lp(a) and LDL-C**

| **Parameter** | **Lp(a) (median, IQR)** | **LDL-C (median, IQR)** | ***P* Value** |
| --- | --- | --- | --- |
| Absolute change (mg/dL) | 0.7 (8.4) | -12 (51.0) | <0.01 |
| Relative change (%) | 6.0 (56.8) | -14.2 (49.7) | <0.01 |
| Correlation with baseline (absolute) | rho=-0.01 (P=0.45) | rho=-0.61 (P<0.01) |  |
| Correlation with baseline (relative) | rho=-0.12 (P<0.01) | rho=-0.54 (P<0.01) |  |

LDL-C, low-density lipoprotein cholesterol; Lp(a), lipoprotein(a); IQR, interquartile range.

**Supplementary table S2. Sensitivity analyses excluding patients based on lipid-lowering medications and hospitalization status**

| **Variable** | **Original**  **OR (95% CI)** | **Excluding Statins**  **OR (95% CI)** | **Excluding Ezetimibe**  **OR (95% CI)** | **Excluding PCSK9 inhibitors**  **OR (95% CI)** | **Excluding Hospitalization**  **OR (95% CI)** |
| --- | --- | --- | --- | --- | --- |
| Baseline Lp(a) | 0.67 (0.63–0.71)* | 0.71 (0.61–0.83)* | 0.73 (0.68–0.79)* | 0.66 (0.62–0.71)* | 0.63 (0.58–0.68)* |
| Follow-up Lp(a) | 1.84 (1.73–1.97)* | 1.86 (1.61–2.16)* | 1.76 (1.63–1.90)* | 1.87 (1.75–1.99)* | 1.92 (1.78–2.07)* |
| BMI | 0.97 (0.95–1.00)* | 0.96 (0.92–1.00) | 0.98 (0.95–1.00) | 0.98 (0.95–1.00)* | 0.97 (0.95–1.00)* |
| Hemoglobin | 1.10 (1.04–1.17)* | 1.09 (0.97–1.24) | 1.11 (1.04–1.19)* | 1.10 (1.04–1.17)* | 1.09 (1.02–1.17)* |
| Platelet count | 1.02 (1.00–1.03)* | 1.02 (0.99–1.05) | 1.02 (1.00–1.03)* | 1.02 (1.00–1.03)* | 1.01 (1.00–1.03) |
| Glucose | 1.03 (1.01–1.05)* | 1.03 (0.97–1.10) | 1.03 (1.01–1.06)* | 1.03 (1.01–1.05)* | 1.03 (1.01–1.06)* |

Multivariable logistic regression was performed with adjustments for age, gender, smoking status, alcohol consumption, socioeconomic status, body mass index (BMI), baseline and follow-up Lp(a), hypertension, diabetes, dyslipidemia, chronic kidney disease, liver disease, thyroid disease, history of myocardial infarction, stroke, heart failure, hemoglobin level, white blood cell count, platelet count, blood urea nitrogen, creatinine, estimated glomerular filtration rate, total cholesterol, LDL-cholesterol, HDL-cholesterol, triglycerides, glucose, aspartate aminotransferase, alanine aminotransferase, total bilirubin, albumin, antiplatelet medication, antihypertensive medication, and antidiabetic medication. *P Values for indicated variables are all <0.05.
